# Cake or broccoli? Recency biases children’s verbal responses

**DOI:** 10.1371/journal.pone.0217207

**Published:** 2019-06-12

**Authors:** Emily Sumner, Erika DeAngelis, Mara Hyatt, Noah Goodman, Celeste Kidd

**Affiliations:** 1 Department of Cognitive Sciences, University of California, Irvine, California, United States of America; 2 Institute of Child Development, University of Minnesota, Minneapolis, Minnesota, United States of America; 3 Department of Brain and Cognitive Sciences, University of Rochester, Rochester, New York, United States of America; 4 Department of Psychology, Stanford University, Stanford, California, United States of America; 5 Department of Psychology, University of California, Berkeley, California, United States of America; Abertay University, UNITED KINGDOM

## Abstract

One of the greatest challenges of developmental psychology is figuring out what children are thinking. This is particularly difficult in early childhood, for children who are prelinguistic or are just beginning to speak their first words. In this stage, children’s responses are commonly measured by presenting young children with a limited choice between one of a small number of options (e.g., “Do you want X or Y?”). A tendency to choose one response in these tasks may be taken as an indication of a child’s preference or understanding. Adults’ responses are known to exhibit order biases when they are asked questions. The current set of experiments looks into the following question: *do children demonstrate response biases*? Together, we show that 1) toddlers demonstrate a robust verbal recency bias when asked “or” questions in a lab-based task and a naturalistic corpus of caretaker-child speech interactions, 2) the recency bias weakens with age, and 3) the recency bias strengthens as the syllable-length of the choices gets longer. Taken together, these results indicate that children show a different type of response bias than adults, recency instead of primacy. Further, the results may suggest that this bias stems from increased constraints on children’s working memory.

## Introduction

One of the greatest challenges of developmental psychology is figuring out what children are thinking. This is particularly difficult in early childhood, for children who are prelinguistic or are just beginning to speak their first words. In this stage, children’s responses are commonly measured by presenting young children with a limited choice between one of a small number of options (e.g., “Do you want X or Y?”). For example, in classical Piagetian conservation tasks [1], children are asked verbally “Does this one have more juice, does this one have more juice, or are they both the same?” A tendency to choose one response in these tasks may be taken as an indication of a child’s preference or understanding.

Adults’ responses are known to exhibit order biases when they are asked questions. For example, adults who are asked to recall items from a list are more likely to remember the first and last items (primacy and recency biases, respectively)[2–4]. Adults have a tendency to choose the first of two presented options (e.g., they choose “heads” when asked, “heads or tails?”)[5,6]. These biases, however, have gone largely unstudied in children.

Understanding whether young children have a bias when responding to questions is important, and not only for the applied purposes of researchers who are designing experiments that involve verbal responses from young children. Understanding whether children exhibit the same or different biases from adults has the potential to inform what we understand about the cognitive processes that underlie language production and decision-making in general.

There’s an additional reason to care about the existence of response biases of young children, which is that verbal productions are typically taken as strong evidence that the child understands at least something about the word they have uttered. For example, studies of children’s language development often use children’s productions as reported by parents using the MacArthur Bates Communicative Development Inventories [7] as children’s lexical knowledge and familiarity. In fact, it is quite tempting for anyone who interacts with small children, not only developmental psychologists, to presume a child’s productions, like an adult’s productions, imply conceptual understanding. But this is not always the case. A toddler who is asked if they threw food “on accident or on purpose,” for example, may respond with one of the two choices without any actual knowledge what either difficult-to-infer abstract concept actually means. If children exhibit a stronger place-based bias than adults, this could indicate that productions of this type are only weak evidence of any underlying understanding of the word’s meaning. In other words, it may make us question whether all productions should be taken strong evidence of word comprehension.

If children exhibit a different type of bias from adults, this could also be informative about what the underlying cognitive mechanisms driving these biases might be. We know that the core cognitive capabilities of young children differ substantially from those of adults. For example, children have lower levels of working memory and inhibitory control [8]. Understanding how these core abilities interact with verbal biases could inform the cause of the biases themselves.

The current set of experiments looks into the following question: *do children demonstrate response biases*? We hypothesize that toddlers, who are just learning language, will be more likely to demonstrate a recency bias when presented with the sentence structure, “X or Y?”. Since younger children possess a limited memory capacity, children could be relying on the phonological loop to remember the option following the word “or”. The phonological loop accounts for recency biases in adults, meaning that during free recall the most recently listed options are more accessible.

Together, we show that 1) toddlers demonstrate a robust verbal recency bias when asked “or” questions in a lab-based task and a naturalistic corpus of caretaker-child speech interactions, 2) the recency bias weakens with age, and 3) the recency bias strengthens as the syllable-length of the choices gets longer. Taken together, these results indicate that children show a different type of response bias than adults, recency instead of primacy. Further, the results may suggest that this bias stems from increased constraints on children’s working memory.

## Experiment 1

### Do toddlers demonstrate recency biases during question answering?

To investigate whether toddlers show any type of bias during question answering, we tested 24 one- to two-year-olds in a game that required them to choose between two (counterbalanced) choices about stickers. In this game, children had to answer several questions with two possible response options (e.g., “Should Rori bring a lunchbox or a backpack to school?”) and were much later (after enough other questions were asked to mask the repetition) again asked the same question with the choice options flipped (“Should Quinn bring a backpack or a lunchbox to school?”).

### Experiment 1 method

#### Participants

Twenty-four children (mean = 2.02 years old, age range = 1.77–2.25 years old) with normal vision and hearing were recruited from the database of volunteer families from the greater Rochester, NY area. Participants were from home environments where they were exposed to at least 90% English. Families were compensated $10 and a child-sized t-shirt for their participation in the study. Six additional children were tested in the study, but were excluded from the final analysis because of failure to complete at least half of the total number of trials (mean = 2.07 years old, range = 1.80–2.17 years old). This research was approved by the University of Rochester Institutional Review Board (Protocol RSRB #43091). All families provided written consent prior to testing. Detailed versions of our protocol and our data are on the Open Science Framework (https://osf.io/yub7c/).

#### Materials

The stimuli consisted of two sets of 20 questions, each containing two choices (see Supporting Information or our Open Science Framework page for the questions). The second set of questions was identical to the first, except that choices were presented in the opposite order. Question choices varied in terms of their commonality and frequency in child-directed speech. Some words were highly familiar to most 2-year-olds (e.g., red, cat) and some were likely to be unfamiliar (e.g., ganache, khaki). Questions also varied in terms of where the choices appeared within them. Some questions contained choices at the end (e.g., “Does Rori like to eat apples or bananas?”), some contained choices at the middle (e.g., “Should Rori bring a backpack or a lunchbox to school”), and some contained choices at the beginning (e.g., “The school has burgers and the school has pizzas. Which one should Rori eat?”).

#### Procedure

**Fig 1:**
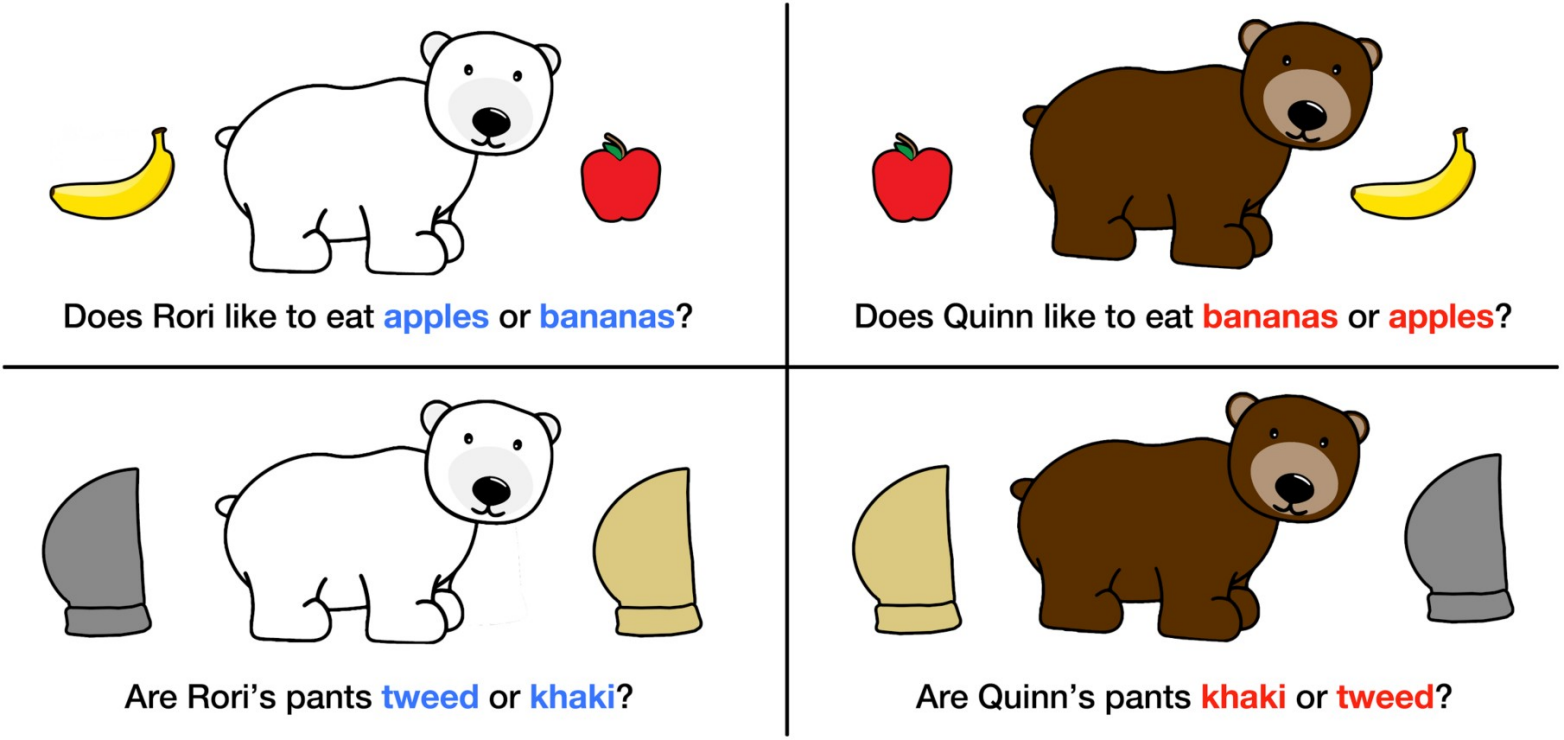
Set up for Experiment 1. Static-cling stickers used in the experiment, along with verbally presented questions. Children were asked a series of questions about each bear–first in one order (e.g., choices in blue on the left) and later in the opposite order (e.g., choices in red on the right). Children were only shown the stickers after they responded to the question verbally.

Children were tested in a quiet testing room without their parents present to prevent parental influence on children’s responses. Testing sessions were videotaped so the data could be coded and entered at a later time. The testing room contained a small, child-sized table with a 17'' x 14'' double-sided whiteboard easel. Participants sat across the table from the researcher who explained that they were going to play a game together. The researcher introduced the child to a bear character (named either Rori, a polar bear, or Quinn, a grizzly bear) whose image was printed on a static-cling sticker and placed on the easel in front of the child. The researcher asked the child if they could help Rori/Quinn make some choices and that for each answer, the child would get a sticker to place on the board (see Fig 1).

The researcher asked the child a set of questions about items for the bear character (e.g., “Is Rori’s shirt red or yellow?”). The stickers corresponding to the options were kept out of view of the participant while the questions were asked verbally. Once participants gave an answer, they were given the static-cling sticker that depicted their choice to stick on the board. The question was repeated one more time if the child did not make a choice after the question was asked the first time. If the child still did not respond, the researcher held up the two corresponding stickers and repeated the question up to two more times. The two stickers were presented in a randomized left right configuration, each equidistant from the child. Children either responded to this final question verbally or by pointing, and were given the appropriate sticker to place on the board.

Once the child ran through the first set of questions, they were asked the same set of questions for the new bear character with the choices presented in the opposite order. For example, if they were asked if Rori’s shirt was “red or yellow” during the first part of the experiment, they were asked if Quinn’s shirt was “yellow or red” during the second part. The order of the question sets was randomized across participants.

#### Coding

Two researchers coded each child’s responses from the video recording of the testing session. Additionally, the child’s verbal skills were ranked on a scale of 1 to 4 independently by two researchers who observed the participant in the waiting room in advance of the study, according to a predetermined set of rating criteria relating to mean utterance length (see S1 File). The two coders assigned the same verbal-skill rating to children 70.83% of the time and differed by one 29.17% of the time. When coders differed, the two verbal-skill rating values for that child were averaged. Child participants had a median verbal-skill rating of 2 (mean = 2.1, range = 1–4).

#### Analysis

Our primary analysis examined the proportion of second-choice responses children made when verbally responding to the questions. This analysis allowed us to determine if children have a bias to respond with the most recently mentioned option. We used a Wilcoxon signed-rank test in order to compare this proportion to the proportion we would expect by chance or preference—*mu* = 0.5. We also compared the proportion of second-choice responses that children made verbally to those that they made non-verbally. If, in fact, children exhibit a recency bias because recent linguistic material is more readily available in their phonological loop, we would expect to see an effect only for verbally answered questions. We used a Wilcoxon signed-rank test because we cannot assume that our data is normally distributed, we are comparing proportions, and it is a more conservative measure than a t-test.

Additionally, we used R and *lme4* to conduct a generalized linear mixed effect analysis in order to evaluate the influence of whether the response was verbal, along with other factors (e.g., age, verbal skills, choice familiarity/frequency, whether or not the choices occurred at the end of the question, and which bear they were answering questions about) on children’s likelihood of responding with the second choice. We used the following as fixed effects: verbal response, age, bear, trial, and frequency of choice words (using the Google Books NGram corpus for English [9]). We used random slopes and intercepts for each of these factors by individual. We included crossed random effects of participant and item pair. The analysis script can be found on our Open Science Framework page (https://osf.io/yub7c/).

### Experiment 1 results

#### Recency biases in verbal responses

**Fig 2:**
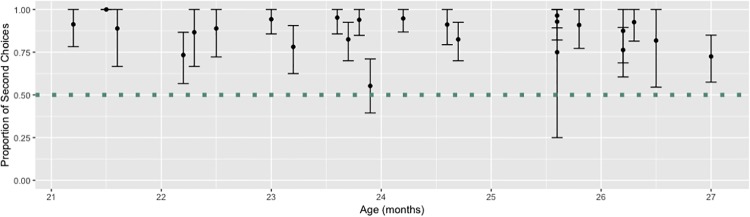
Robust recency bias across ages in Experiment 1. Each dot represents an individual’s proportion of second choices during the trials in which they responded verbally. The error bars depict 95% confidence intervals. The dotted line represents chance. Twenty-two of the twenty-four participants exhibited significantly more second-choice responses than would be predicted by chance.

Overall, when participants responded verbally, they chose the second choice 85.2% of the time—a value that a Wilcoxon signed rank test confirmed to be significantly above chance (V = 231, *p* < .0001). Further, 22 of the 24 subjects showed significantly more second-choice responses than chance would have predicted in an analysis of individual subjects (Fig 2).

Participants responded verbally 78.7% of the time. When looking at trials where children responded by pointing, participants chose the second choice only 51.6% of the time, which was not significantly different from chance (*V* = 102, *p* = .79). The proportion of second choices was significantly higher for verbal than non-verbal responses (*W* = 384.5, *p* < .0001). This comparison of verbal to nonverbal responses suggests that this bias is limited to the verbal domain.

In the generalized linear mixed effect model, we found that when the participant responded verbally, the chance of choosing the second option increases (β = 2.13, *p* = 9.6e-12). We also found a small effect of age, suggesting that the younger participants are more likely to respond with the second option (β = -0.29, *p* = .02).

Verbal skills, choice familiarity/frequency, sentence structure, and the bear they were answering questions about did not reach significance (see Table 1 for more detail).

**Table 1:** Experiment 1 generalized linear mixed effect model results.

Factor	Coef.	SE	*z*	*p*
*Intercept*	3.52	1.93	1.82	.07*
VerbalResponse(y)	2.13	0.31	6.81	9.592e-12***
scale(AgeMo)	-0.29	0.13	-2.23	.02*
Trial	0.00	0.02	0.31	.75
VerbalSkill	0.03	0.17	0.22	.82
ChoiceLocationEnd	0.21	0.36	0.58	.56
ChoiceLocationMid	-0.28	0.43	-0.66	.50
scale(log(Freq))	-0.06	0.12	-0.45	.65
Bear	-0.11	0.51	-0.22	.83

* < .1

** < .05

*** < .005

### Experiment 1 discussion

In Experiment 1, we show that toddlers exhibit a robust recency bias when responding verbally to “or” questions. Since this bias appears to be limited to the verbal domain, it suggests that the cause of this bias may be working memory constraints.

## Corpus analysis

### Are recency biases present in natural parent child speech?

Experiment 1 found that one- to- two-year-olds are more likely to choose the second option when presented with an “or” question. Do these types of sentences exist in naturalistic speech? Or is our finding limited to the laboratory setting? To determine if the same bias applies in real-world child-parent interactions, we further explore Experiment 1’s findings through analyzing naturalistic parent-child interactions.

### Corpus analysis method

The Child Language Data Exchange System or CHILDES [10] is an online databank of transcribed conversations between parents and their children. We limited our analysis to CHILDES transcript files that involved two participants—one adult and one child—and that included the age of the child. This way we could ensure that questions we extracted from adult speech were most likely directed to a child of a known age (as opposed to a sibling or other adult). From this predefined subset, we extracted 534 two-alternative choice questions. The questions were posed to children ranging in age from 0.74 to 4.54 years of age (mean = 2.51). We then coded children’s responses to the question for whether they chose the first option, the second option, or neither option (e.g., by failing to respond, or responding with an irrelevant verbal response). Of all of the two-choice questions adults asked children in CHILDES, children responded with the first or second option 58.17% of the time (as opposed to not responding or providing an irrelevant response). We then further limited our analysis to only the questions where the child responded with an appropriate responses option, bringing the age range to 1.59–4.39 years of age (mean = 2.68). The final data set included data from the following corpora: Bates [11], Bernstien [12], Demetras [13], Higginson [14], Kuczaj [15], MacWhinney [16], New England [17], Providence [18], Rollins [19], Sachs [20], Snow [21], Suppes [22], Tardif [23], and Van Houten [24]. The dataset used, as well as the script we used to extract the data from CHILDES is openly available on the Open Science Framework (https://osf.io/yub7c/).

### Corpus analysis results

#### Recency bias present natural parent-child setting until around age three

**Fig 3:**
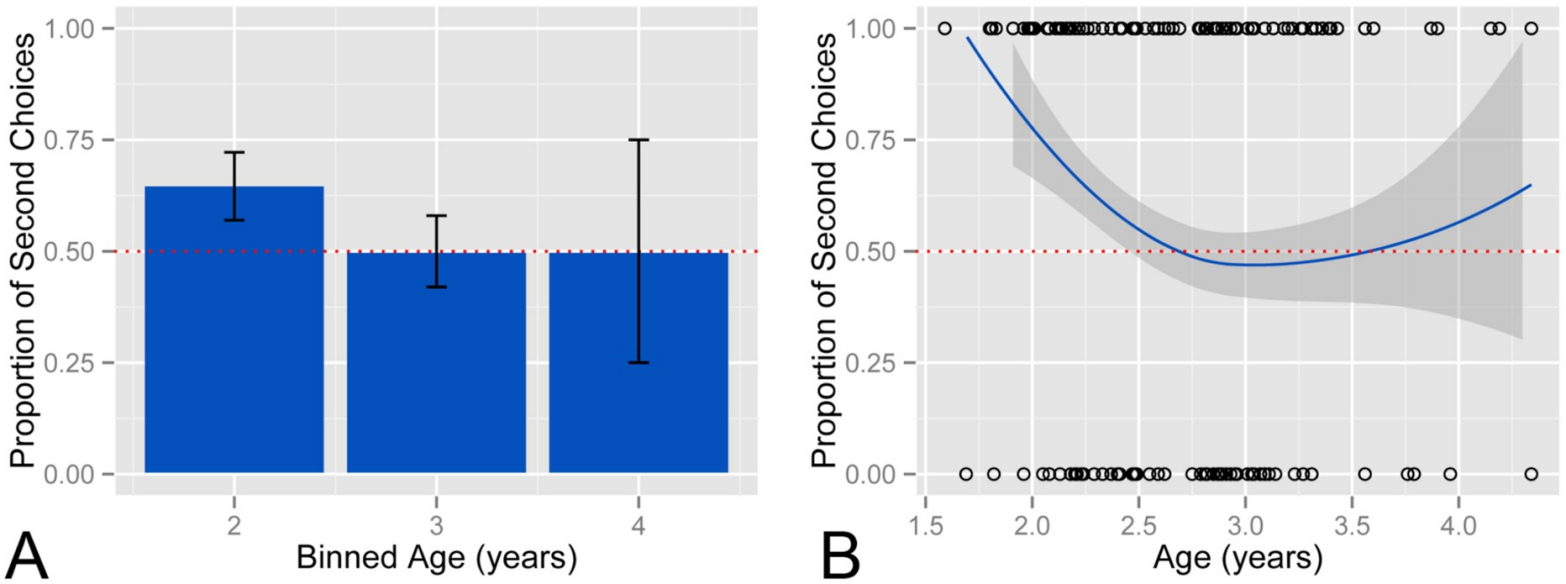
Recency bias present in natural parent-child setting. (A) Proportion of second-choice responses in CHILDES binned by age of the children in years. (B) A smoothed LOESS curve fitted to the raw data from CHILDES (empty dots).

A linear regression suggests that as children get older, the likelihood of choosing the second option decreases (*t* = -2.65, *p* = .009). The proportion of first and second choice responses children gave binned by age appears in Fig 3A. As evident from the plot, binned responses from 2-year-olds are significantly more biased towards the second-choice option, as compared to chance. This bias is no longer significant in the 3- and 4-year-olds, who are equally likely to select the first and second choices. Fig 3B shows the raw data (displayed as circles at the top and bottom of the plot to represent the 1’s and 0’s encoding whether each response was for the second-choice option), as well as a LOESS curve fitted to the data. The smoothed fit to the data suggests that young children exhibit a strong second-choice bias, but that choices are at chance starting at between 2.5 and 3 years of age. The data suggests that this recency bias persists until around the third year of life.

### Corpus analysis discussion

Here, we found further evidence that children under the age of 3 are biased towards choosing the second response option when faced with the sentence structure, “X or Y?”. Interestingly, this bias seems to disappear with age. Unlike Experiment 1, where parents are not present in order to minimize parental bias, the Corpus Analysis only looks at conversations between parents and their children. Thus, these results may be confounded by nonverbal cues which older children may be better at picking up than younger children. Regardless, these results can be considered a conceptual replication of the results we found in Experiment 1 because the recency bias is still present in children under the age of 3.

## Experiment 2

Experiment 1 and the Corpus Analysis demonstrate that toddlers are more likely to choose the second response option when making choices verbally, both in laboratory and naturalistic settings. A number of things could be the underlying causes of this bias. For one, younger children might be more sensitive to the prosodic stress and lengthening at the end of an utterance [25]. While this fact could certainly contribute to the overall effect, it cannot be the primary driver because the recency bias was still robust even when choices occurred in the middle of an utterance (e.g., “Should Rori bring a cat or a dog for show and tell?”). Another possibility could be the fact that younger children possess a more limited working memory capacity [8]. The last-mentioned option could simply be the only one available in their phonological loop.

In Experiment 2, we test the hypothesis that working memory limitations underlie the recency bias. We created a task with novel words for preschool aged children who have more advanced language skills. Twenty-four 3- to 4-year-old children participated in a counterbalanced, binary choice toy naming game. In this game, children were asked to help the experimenter decide what to name toy characters (e.g., “Should we name this toy Stog or Meeb?”). The name options represented novel words that vary in syllable length (from 1 to 4 syllables). After naming all of the toys, they would be asked to name the toys again with the position flipped. Additionally, we evaluated working memory abilities through a syllable repetition task [26].

If working memory limitations are responsible for the previously observed recency bias, we would expect to observe more of a recency bias for novel words that have greater syllable lengths. Further, we would expect for children who perform worse on the syllable repetition task to exhibit more of a recency bias than peers who perform better on the task.

### Experiment 2 method

#### Participants

Twenty-four children (3.06 yo– 4.97 yo) children were tested in this study. The children were recruited from a database of volunteer families from the greater Rochester, NY area. All participants had normal vision and hearing according to parental report. They were also from homes where they were exposed to at least 90% English. Families were compensated $10 and a child-sized t-shirt for their participation in the study. One additional child was excluded from the final analysis because of failure to complete at least half the trials in the study. This research was approved by the University of Rochester Institutional Review Board (Protocol RSRB #54944). All families provided written consent prior to testing.

#### Materials

The visual stimuli consisted of 20 pictures of toy characters. These are photos we took of small art collectible figures (the photos can be found on our Open Science Framework page: https://osf.io/5vdqm/). We chose these images because they are child friendly, and it is unlikely that our participants have encountered any of them before. The order of the toy character presentation was fixed. Two counterbalanced sets of 20 questions in a randomized order were presented. We randomized the question order to control for any preferences and variation in the visual saliency of the photos. Toy name options were varied in word length up to four-syllables long. There were five instances of each syllable length per set (See the S2 File for the list of questions.).

#### Syllable repetition task (SRT)

After the main task, children completed a syllable-repetition task to evaluate working memory through the methods outlined in Shirberg & Lohmeier [26]. In this task, children were told to repeat back words that vary from two-to-four syllables. Children were scored on the number of consonants they say correctly since the vowels stay the same throughout the task (e.g., bama, bamana, and bamadana). There were 18 different words in total that they were asked to repeat back.

#### Procedure

**Fig 4:**
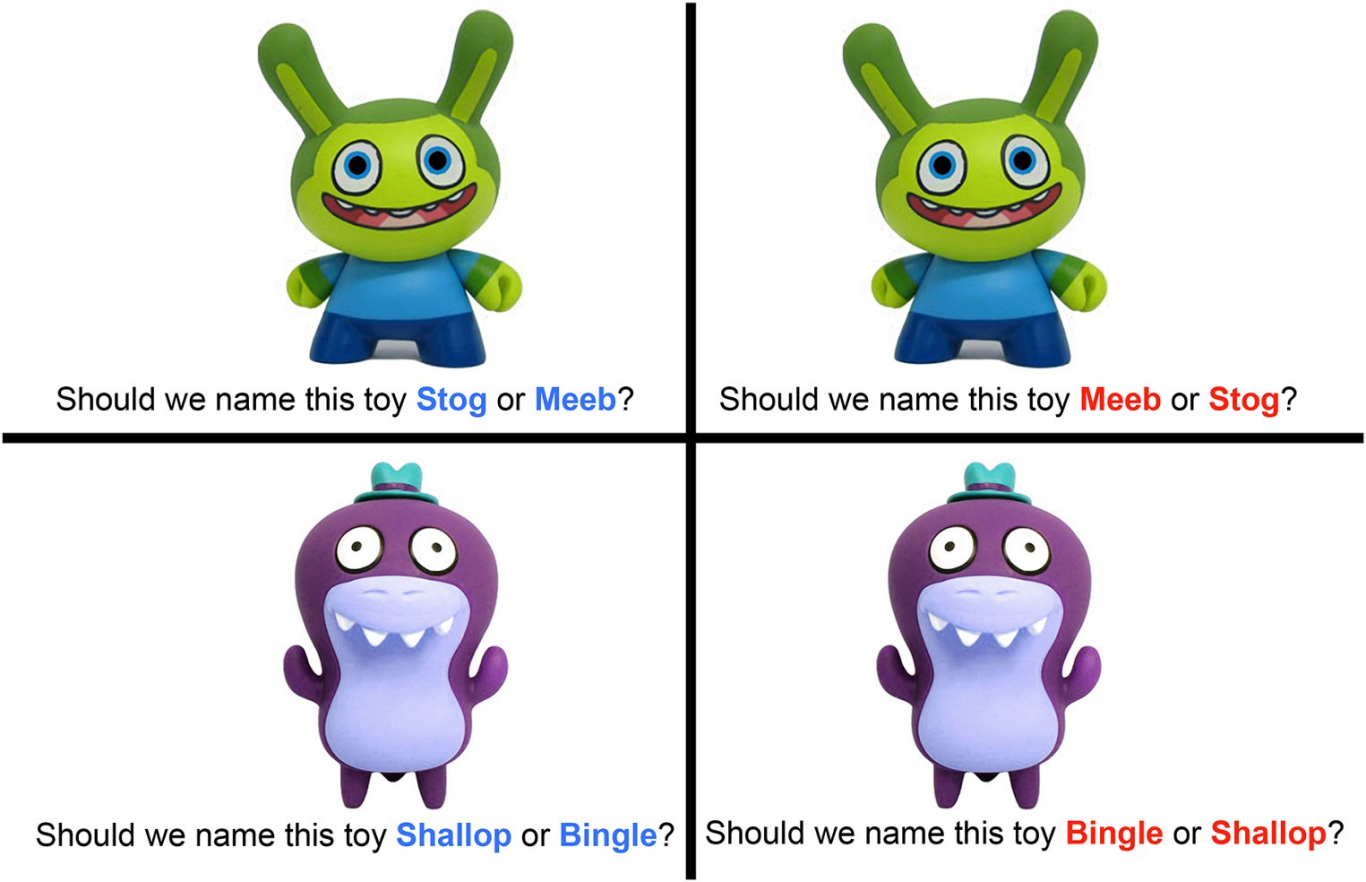
Set up for Experiment 2. Examples of novel toy pictures used in the experiment, along with the verbal prompt. Children were asked to name a series of 20 toys (e.g., choices in the blue on the left). Later they were shown the same toys and asked to name them again in the opposite order (e.g., choices in the red on the right). Names varied in syllable length.

Participants were tested in a quiet room without their parents present. Testing sessions were videotaped so the data could be entered at a later time. Children were invited to sit opposite the researcher at a small table. Children were then asked to help the researcher name toys. Participants were shown pictures of novel cartoon characters and asked to decide between two “names” to give the toy (e.g., “Should we name this toy Stog or Meeb?”). Pictures were presented in an 8” by 8” flipbook (Fig 4). Each question contained two novel words that matched in number of syllables. If the child did not respond after two repetitions, the trial was not included in the final analysis and the experimenter moved to the next picture. Our procedures, materials, and data are available on the Open Science Framework (https://osf.io/yub7c/).

#### Analysis

Our analysis looked at the proportion of second-choice responses children made to see if children were demonstrating any sort of bias towards the first or last option. To do this, we used a Wilcoxon signed-rank test to compare the proportion of second-choice responses to that predicted by chance (mu = .50).

Additionally, we used R and *lme4* to conduct a generalized linear mixed effect analysis in order to evaluate the influence of whether word length, age, and performance on the syllable repetition had an effect on children’s likelihood of responding with the second choice. We used fixed effects of word length, age, and SRT performance. We used random slopes and intercepts for each of these factors by individual. Additionally, we added crossed random effects of individual and name pairing. Code for this analysis can be found on our Open Science Framework page (https://osf.io/yub7c/).

### Experiment 2 results

#### Recency bias present in preschoolers

**Fig 5:**
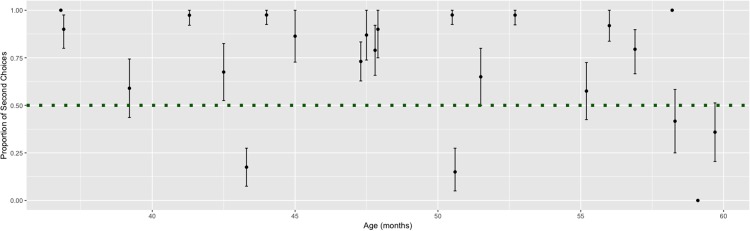
Recency bias present in preschoolers. Each dot represents an individual’s proportion of second choices. The error bars depict 95% confidence intervals. The dotted line represents chance. Fourteen participants exhibited significantly more second-choice responses than chance would predict. Three participants exhibited significantly less second-choice responses than chance would predict.

We found that on average, children demonstrated a recency bias (See Fig 5). A Wilcoxon signed-rank test revealed that the proportion of second choice selections is significantly higher than would be predicted by chance (*V* = 248, *p* < .006). However, when looking at individual participants’ performance, we found that 14 participants demonstrated a recency bias (e.g., selecting significantly more second-choice responses) and 4 participants demonstrated a primacy bias (e.g., selecting significantly more first choice responses, as adults tend to do given binary choices).

#### The recency bias is stronger when the words are longer

**Fig 6:**
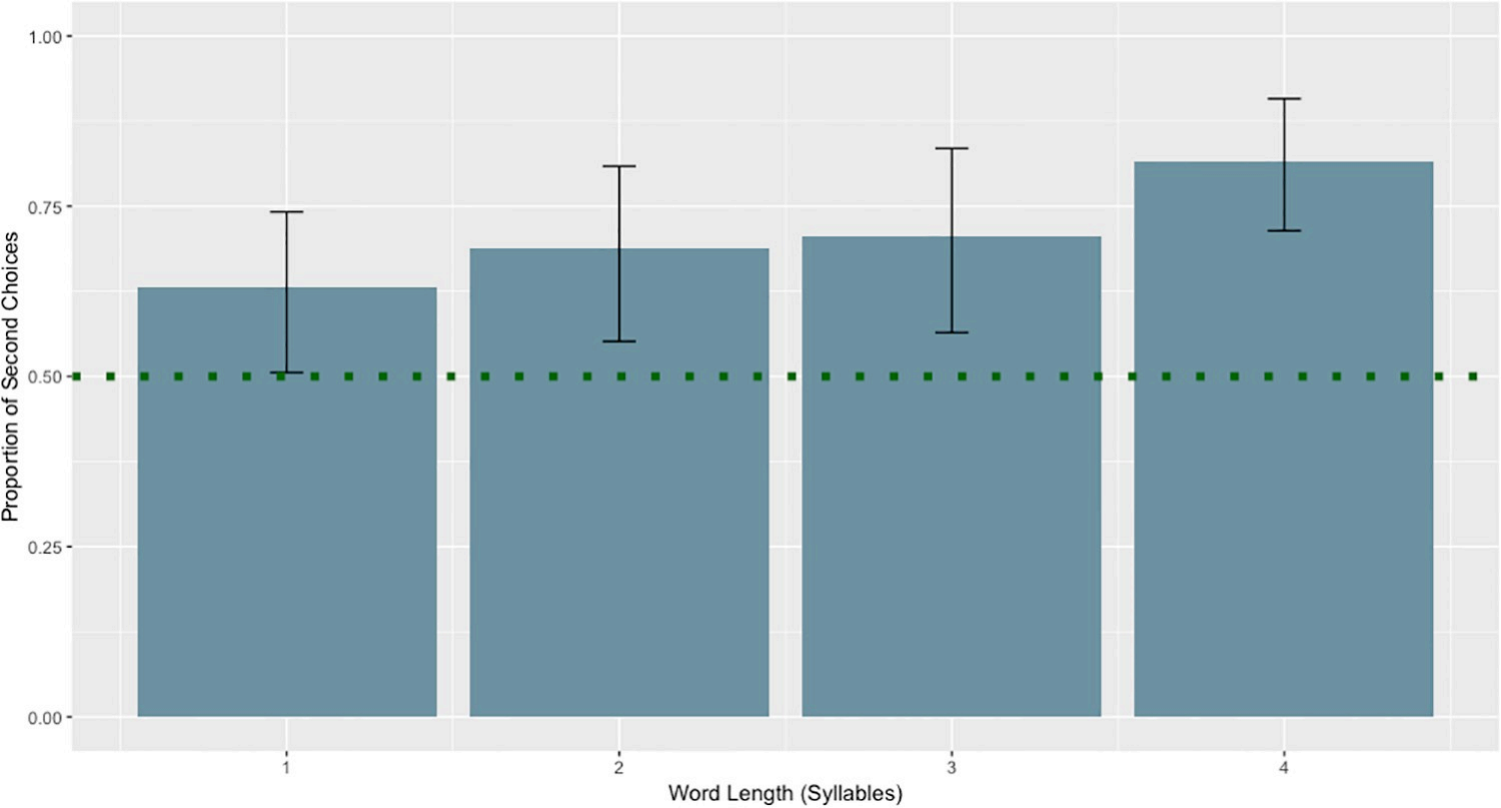
Bias is stronger when choice options have more syllables. Proportion of second choices made separated by word length. Dotted line indicates chance. For words two syllables or more, the proportion of second choice selections is significantly above chance.

We used R and *lme4* to conduct a generalized linear mixed effect analysis. We used age, number of syllables in the response options, and performance on the syllable repetition task as fixed effects. This revealed that the number of syllables in the name was a significant predictor of second-choice responses (see Fig 6 and Table 2). Longer words had more second-choice responses (β = 0.47, *z* = 4.18, *p =* 2.9e-5). As children got older, performance on the working memory measure (Syllable Repetition Task, or SRT) increased (r = 0.49, *p* = 1.07e-6). However, age & performance on the SRT were not predictors of choosing the second item.

**Table 2:** Experiment 2 generalized linear mixed effect model.

Factor	Coef.	SE	*z*	*p*
*Intercept*	2.88	4.63	0.62	.53
Syllables	0.47	0.11	4.18	2.9e-5***
Centered age	-0.36	0.81	-0.45	.654
SRT	0.11	0.44	-0.24	.81

* < .1

** < .05

*** < .005

### Experiment 2 discussion

Here, we show that preschoolers exhibit a similar recency bias when they are given two nonsense words to choose between verbally. We found that this bias was stronger when the response options were longer, which suggests that working memory constraints may be the underlying mechanism of this bias. However, we found that age and performance on the Syllable Repetition Task were *not* predictors of participants choosing the second response option, thus more work on this topic would be necessary to conclude this.

## Discussion

We have found that young children demonstrate a robust recency bias when answering two-alternative forced-choice questions verbally. This recency bias can be seen both in the laboratory and within naturalistic contexts. The bias decreases with age, but returns when response options are novel and have more syllables. The response bias we have identified has many implications, both theoretically and practically, in how we evaluate young children’s lexical knowledge during early stages of speech production. Additionally, our findings shed light on how we view the connection between decision-making biases and working memory.

### Theoretical implications

Although it is known that young language learners utilize repetition during the early stages of language acquisition (e.g., echolalia), what is novel about our findings is that imitation alone cannot describe our results. In fact, it has been posited that the purpose of the phonological loop is to help us learn new words, particularly in its ability to enable imitation, which is a common phenomenon during the early stages of language learning. Indeed, this simple strategy could be very useful in enabling toddlers to engage in verbal exchanges before they possess fully developed semantic representations for the words that they are using.

It’s important to note here, however, that the recency bias was present throughout all of Experiment 1, even when the response options were embedded in the middle of the question (e.g., “Should Rori bring a lunchbox or a backpack to school”). This rules out the possibility that children are simply repeating back the last option that they hear. The phonological loop could still be involved, but the mechanism must be a bit more sophisticated to account for the data from non-utterance-final choices across our experiments. Children must recognize the word “or” separates choices, and be able to identify and hold on to the second item until the moment of their response. We note that previous work has questioned whether children understand the meaning of the word “or” on the basis of children sometimes mistaking the meaning of disjunctives (e.g., “or”) to be a conjunctive (e.g., “and”) [27]. For example, preschoolers may interpret the sentence, “Every boy is holding an apple or a banana” as “every boy is holding an apple and a banana.” While we saw no evidence that children struggled with the meaning of the word “or” in our study, this difference could be due to the visual pragmatics of the task, which may have more readily suggested to children that the choice was one or the other, not both.

In any case, our results, particularly the word-length effect we found in Experiment 2, suggests that this bias may be related to children’s limited working memory capacity. However, we did not find an effect of age and working memory in Experiment 2. Perhaps if our age range was larger and if we didn’t do the syllable repetition task directly after the toy naming task, we would have found an effect of working memory or age.

While we focused our studies on children, who are known to have limited working memory capacities, the same effects would be expected to apply to anyone with sufficiently taxed working memory. In adults, conditions that impair working memory (e.g., sleep deprivation, inebriation, multitasking, disorders associated with working memory deficits) would all be expected to result in similar recency biases. In other words, these results shed light not only on children’s verbal behavior, but more generally on memory and response biases.

### Practical implications

Modern studies of language development commonly assume that children’s lexical productions are indicators of their lexical knowledge. The production norms in the MacArthur-Bates Communicative Development Inventories [7] are commonly used to estimate children’s comprehension abilities throughout development. However, our findings suggest that some of children’s earliest of utterances may be the result of a recency bias, and do not necessarily indicate that the child comprehends the words they speak. Thus, data collected via vocabulary surveys like the MacArthur-Bates Communicative Development Inventories may be an overestimation of children’s true word knowledge.

Additionally, researchers working with this age group should take special care with any experimental methodologies that rely on children’s verbal responses. Methods that require verbal responses should ensure question choices are carefully counterbalanced. Parents, however, may wish to use such a biased design: “Would you like cake or broccoli?”

## Supporting information

S1 File Questions for Study 1. The questions for Study 1 can be found here.
(DOCX)

S2 File Names for Study 2. The names for the questions in for Study 2 can be found here.
(DOCX)
