# Supplementary material for: Cake or broccoli? Recency biases children’s verbal responses
Source: PLoS One. 2019 Jun 12;14(6):e0217207. doi: 10.1371/journal.pone.0217207 (PMC6561545; doi:10.1371/journal.pone.0217207)
Supplement: S1 File — The questions for Study 1 can be found here. (DOCX) [file pone.0217207.s001.docx]

**Study 1 Questions**

**Note – half of participants started with Set A. The other half started with Set B.**

**SET A QUESTIONS**

Should Rori bring a **backpack** or a **lunchbox** to school?

Does Rori like to eat **apples** or **bananas**?

Should Rori use a **brush** or a **comb**?

Does Rori live in an **igloo** or a **teepee**?

Rori has a **hat**. Rori has a **cap**. Which should Rori wear?

Should Rori bring a **cat** or a **dog** for show and tell?

The school has **burgers**. The school has **pizzas**. Which should Rori eat?

Does Rori like **yogurt** or **cereal**?

Are there **crocuses** or **tulips** in the park?

Does Rori like **apple** **juice** or **orange** **juice**?

Are Rori’s pants **khaki** or **tweed**?

Rori has a **green** **toy**. Rori has a **purple** **toy**. Which does Rori like?

Does the playground have **pine** or **oak** trees?

Is the school fence **brick** or **wood**?

Is Rori’s shirt **yellow** or **red**?

Rori has a **bike**. Rori has a **scooter**. Which should Rori ride?

Are the cupcakes **ganache** or **red** **velvet**?

Does Rori wear **sandals** or **sneakers**?

Should Rori play **football** or **basketball**?

Does Rori like **grape** or **lime** candy?

**SET B QUESTIONS**

Should Quinn bring a **lunchbox** or a **backpack** to school?

Does Quinn like to eat **bananas** or **apples**?

Should Quinn use a **comb** or a **brush**?

Does Quinn live in a **teepee** or an **igloo**?

Quinn has a **cap**. Quinn has a **hat**. Which should Quinn wear?

Should Quinn bring a **dog** or a **cat** for show and tell?

The school has **pizzas**. The school has **burgers**. Which should Quinn eat?

Does Quinn like **cereal** or **yogurt**?

Are there **tulips** or **crocuses** in the park?

Does Quinn like **orange** **juice** or **apple** **juice**?

Are Quinn’s pants **tweed** or **khaki**?

Quinn has a **purple** **toy**. Quinn has a **green** **toy**. Which does Quinn like?

Does the playground have **oak** or **pine** trees?

Is the school fence **wood** or **brick**?

Is Quinn’s shirt **red** or **yellow**?

Quinn has a **scooter**. Quinn has a **bike**. Which should Quinn ride?

Are the cupcakes **red** **velvet** or **ganache**?

Does Quinn wear **sneakers** or **sandals**?

Should Quinn play **basketball** or **football**?

Does Quinn like **lime** or **grape** candy?
