# Supplementary material for: Cake or broccoli? Recency biases children’s verbal responses
Source: PLoS One. 2019 Jun 12;14(6):e0217207. doi: 10.1371/journal.pone.0217207 (PMC6561545; doi:10.1371/journal.pone.0217207)
Supplement: S2 File — The names for the questions in for Study 2 can be found here. (DOCX) [file pone.0217207.s002.docx]

**STUDY 2**

**Note – half of participants started with Set A. The other half started with Set B.**

**SET ONE**

Should we call this toy X or Y…

1. Feb Rah
2. Woolakey Fubocket (foo-bock-it)
3. Muktoop Tiffin*
4. Stog Meeb
5. Cagamosis Salmagundi
6. Hepokot Gibbertripe
7. Hootamawhirl Chirotonsor
8. Bingle* Shallop*
9. Cacoethes Sockdolager*
10. Lup Nie
11. Bamboosh Peever*
12. Lampoon* Shmooget
13. Jumbuck* Higgler*
14. Perniscle Riffleguff
15. Kerfuffle* Prattlebok
16. Koob Yeel
17. Jamelot* Bunkhooey*
18. Huskimation Taypermefa
19. Haykidosi Pecadilloes *
20. Wah Pru

**SET TWO**

1. Rah Feb
2. Fubocket Woolakey
3. Tiffin* Muktuk*
4. Meeb Stog
5. Salmagundi Cagamosis
6. Gibbertripe Hepokot
7. Chirotonsor Hootamawhirl
8. Shallop* Bingle*
9. Sockdolager* Cacoethes
10. Nie Lup
11. Peever* Bamboosh
12. Shmooget Lampoon*
13. Higgler* Jumbuck*
14. Riffleguff Perniscle
15. Prattlebok Kerfuffle*
16. Yeel Koob
17. Bunkhooey* Jamelot*
18. Cagamosis Huskimation
19. Pecadilloes * Haykidosi
20. Pru Wah

**1 Syllable Pairs**

1, 21

4, 24

10, 30

16, 36

20, 40

**2 Syllable Pairs**

3, 23

8, 28,

11, 31

12, 32

13, 33

**3 Syllable Pairs**

2, 22

6, 26

14, 34

15, 35

17, 37

**4 Syllable Pairs**

5, 25

7, 27

9, 29

18, 38

19, 39

* indicates real word that children are unlikely to have heard

First choice, second choice

randomize the order
